# Carbon dot-modified silver nanoparticle electrochemical sensors for the ultrasensitive detection of total malachite green and leucomalachite green residues in fish

**DOI:** 10.1039/d6ra01261b

**Published:** 2026-03-30

**Authors:** Pomi Bi Boussou Narcisse, Aka Alla Martin, Essy Kouadio Fodjo, Guangxin Yang, Cong Kong, Zhen Gu, Koffi Koffi Kra Sylvestre, Irié Bi Irié Williams

**Affiliations:** a Laboratory of Constitution and Reaction of Matter, UFR SSMT, Felix Houphouet Boigny University 22 BP 582 Abidjan 22 Cote d’Ivoire; b East China Sea Fisheries Research Institute, Chinese Academy of Fishery Sciences Shanghai 200090 P. R. China; c Department of Automation, East China University of Science and Technology 130 Meilong Road Shanghai 200237 P. R. China

## Abstract

Owing to its low cost and high effectiveness as a biocide in aquaculture, malachite green (MG) and its residue leucomalachite green (LMG) are commonly found in aquatic resources. However, they are known to be hazardous contaminants to both the ecosystem and humans. Their side effects on the environment threaten fishery resources and food safety. In this perspective, designing a suitable tool for the quantitative and real-time detection of MG and LMG in foodstuffs is extremely crucial. Herein, we report an ultrasensitive differential pulse voltammetry (DPV) method using a gold (Au) electrode modified with a mixture of silver nanoparticles and carbon dots (AgCDs) to enhance its catalytic properties for the redox reaction of MG and LMG. Carbon dots (CDs) ecologically synthesized from pineapple peel juice *via* a hydrothermal method were used as reducing and stabilizing agents for the synthesis of silver nanoparticles (AgNPs). Besides the resulting AgCDs solution used to modify the Au electrode *via* immersion, a portable electrochemical workstation was used, making field monitoring and real-time detection easier. The synergistic effect of Au and AgCDs makes the modified AgCDs/Au electrode highly effective for the trace detection of total MG and LMG residues. Under optimized parameters and in a Na_2_SO_4_ electrolyte solution, a linear range from 6 pg mL^−1^ to 100 pg mL^−1^ with a detection limit of around 5 pg mL^−1^ was obtained. Furthermore, its precision, accuracy, and application studies on catfish, carp, red carp, and sheatfish suggest that the AgCDs/Au electrode can be used to monitor and determine traces of these contaminants in foodstuffs.

## Abstract 2

Green approach synthesis of silver nanoparticle-covered carbon dots led to ultrasensitive sensor by modifying Au electrode. This sensor was applied for detection of total malachite green and leucomalachite green residues with low limit of detection.

## Graphical abstract

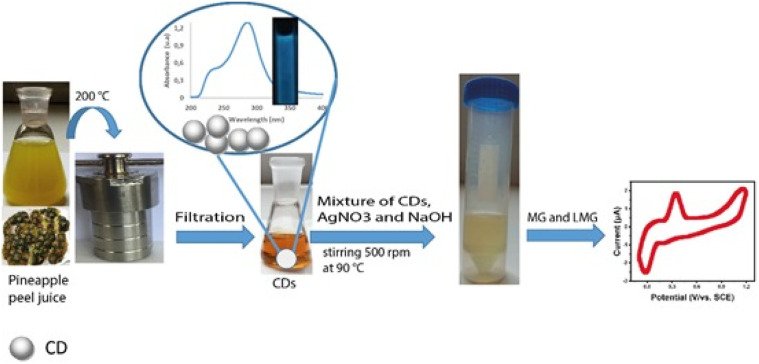

## 1 Introduction

Malachite green (MG), an industrial triphenylmethane cationic dye, is mostly used in dyeing jute, leather, paper, and polymers and especially in aquaculture due to its effectiveness against fungal and parasitic infections in fish.^1^ However, numerous studies have shown that MG and its leucomalachite green (LMG) residue are toxic to mammalian cells and can have teratogenic, carcinogenic and mutagenic effects.^2–4^ Indeed, after penetrating the bodies of aquatic animals, MG is metabolized to highly toxic liposoluble LMG.^5,6^ In addition, as MG is found to be persistent in the environment,^7^ both MG and LMG have been banned or strictly restricted in aquaculture in many countries. For instance, Canada banned MG as a peach fungicide in 1992, while in 1993, the US Food and Drug Administration (FDA) stipulated that the use of MG as a peach fungicide was prohibited. In view of harmonising controls in the EU, a reference point for action (RPA) for the sum of residues of MG and LMG is set to 2 µg kg^−1^ (regulation 2002/675/EC).^8^ In a scenario where MG/LMG residues would only be present in fish and shellfish, a toxicological limit of quantification (TBLOQ) of 0.15 or 0.30 µg kg^−1^ of food (based on the exposure of a toddler or adult, respectively) would serve as a basis for establishing limits.^8^ Besides, Japan has clearly stipulated that there must be no MG residues in aquatic products imported under the positive list system,^9^ while in China, MG has been listed as a banned chemical according to the agricultural industry standard guideline: ‘NY5071-2002 Guidelines for the Use of Pollution-Free Food and Fish Drugs’.^5^

Although MG has been banned in many countries, its use remains common, mainly in African countries where there is no strict regulatory control.^10^ Therefore, developing rapid and ultra-sensitive detection methods for MG and LMG is important to preserve ecological sustainability and food safety. Several methods including chromatography methods have been used as standard methods in laboratories, but these methods are not suitable for on-site analysis due to the equipment size, expensive instrumentation, lengthy procedure of analysis, and especially, the need for professionals with specific skills and related training;^11,12^ hence, spectroscopy-based methods such as fluorescence^13^ and surface-enhanced Raman scattering (SERS)^11,14^ have been used. Although the spectroscopic techniques are simple and very sensitive, their anti-interference capability in complex matrices remains limited. Related researches (Wet NH_3_-Triggered NH_2_-MIL-125(Ti) Structural Switch for Visible Fluorescence Immunoassay Impregnated on Paper; Bioresponsive Release System for Visual Fluorescence Detection of Carcinoembryonic Antigen from Mesoporous Silica Nanocontainers Mediated Optical Color on Quantum Dot-Enzyme-Impregnated Paper widely used in rapid inspection area has significant drawbacks, such as problem of labor intensity and the requirement for enzyme-linked antibodies.^15,16^

The electrochemical method is an attractive alternative for sensing due to its advantages in terms of sensitivity, simplicity, low cost, and easier miniaturization. Assays such as smartphone-based electrochemical immunoassay for the point-of-care detection of SARS-CoV-2 nucleocapsid protein and bimetallic single-atom nanozyme-based electrochemical–photothermal dual-function portable immunoassay with smartphone imaging were successfully investigated. Different materials for the electrode surface modification have been widely used to improve the sensitivity and chemical specificity of electrochemical sensors.^2,17^ However, the macroscopic scale of most crystalline products results in the poor reproducibility of electrocatalytic responses, and the methods used to synthesize these materials are costly, tedious, and potentially toxic to the environment. It has been demonstrated that crystal facet engineering can modulate electron transfer mechanisms by self-powering a photoelectrochemical sensing platform for the noninvasive detection of uric acid. Along the same lines, an environmentally friendly, less expensive synthesis strategy of silver nanomaterials capped with carbon dots (AgCDs) has been proposed for electrode surface modification. These AgCDs are advantageous, as carbon dots can facilitate electron transfer through their surface functional groups.^18^ Based on these considerations, a novel electrochemical sensing platform for malachite green and leucomalachite green detection was developed. The proposed method combines simplicity, miniaturization and portability, without requiring electricity for on-site measurements, which is important for rapid monitoring.

## 2 Materials and methods

### 2.1 Reagents

All chemicals used were of analytical grade, and were used as received without further purification. The following chemicals were used throughout this work. Cod liver oil, serum albumin (99%), acetonitrile (CH_3_CN, HPLC grade), magnesium sulfate (MgSO_4_, 99%), alumina (Al_2_0_3_, 99%), protein, and lipid were obtained from Standard Process Inc., Lac Global Brands (Malaysia), while silver nitrate (AgNO_3_, 99%), malachite green (C_23_H_25_N_2_Cl, 98%), leucomalachite green (C_23_H_26_N_2_, 98%), chloramphenicol (C_11_H_12_O_5_N_2_Cl_2_, 98%), and 4-(dimethylamino)phenol hydrochloride (C_8_H_12_ClNO, 98%) were provided by Alta scientific Ltd (Tianjin, China), and sodium sulfate (Na_2_SO_4_, 99%) was purchased from Sigma-Aldrich (St. Louis, MO, USA). Ultrapure water obtained from a deionized (DI) water system (Sichuan Zhuoyue Water Treatment Equipment Co, Ltd, Chengdu, China) with a resistivity of 18.25 MΩ.cm was used throughout the experiments. Farmed fish samples were collected from a wholesale market. The samples were collected from Gouro markets in Adjame and Cocovico markets in Cocody, two quarters of Abidjan city (Ivory Coast).

### 2.2 Equipment

Scanning electron microscopy (SEM) images were acquired using a SEM FEG Supra 40VP Zeiss (Oberkochen, Germany) equipped with an EDS for the analysis of the elemental composition of the synthesized nanomaterials, and high-resolution transmission electron microscopy (HR-TEM) images were recorded using a Tecnai G2F30 instrument (FEI, USA). Fourier transform infrared (FT-IR) spectra were recorded using a Nicolet NEXUS 670 spectrophotometer (Nicolet, USA). For dynamic light scattering (DLS) analysis, samples of the synthesized CDs or AgCDs were collected and a Zetasizer Nano ZS (Malvern Instruments, UK) was used, and measurements were obtained using a He–Ne laser (633 nm). The zeta potential of the samples was also analyzed using the same Zetasizer Nano ZS equipment. The X-ray powder diffraction (XRD) pattern was recorded using an X′-Pert Diffractometer (Philips Netherlands). All electrochemical measurements were carried out using a MiniOne electrochemical workstation (plug and play style), a homemade equipment provided by the East China University of Science and Technology (MiniOne Instruments, Shanghai, China). A conventional three-electrode system consisting of a modified gold electrode (AgCDs/Au) as the working electrode (*ɸ* = 1 mm), a carbon wire as the counter electrode, and a saturated calomel electrode (SCE) as the reference electrode were used. Before use, the Au electrodes were cleaned using an ultrasonic cleaner (Bandelin electronic super AK 255, Germany). All experiments were conducted at room temperature.

### 2.3 Fabrication of the CDs/Au and AgCDs/Au electrodes

#### 2.3.1 Synthesis of carbon dots (CDs)

To synthesize the CDs, 100 g of pineapple peel was ground in 150 mL of deionized water and then filtered as described in the literature.^19^ Twenty-five milliliters of this filtrate was poured into a 50 mL hydrothermal reactor and heated in an oven at 200 °C for 3 h. A fluorescent brown solution of carbon dots was then obtained. After cooling to room temperature, the mixture was centrifuged at 5000 rpm for 15 min, and then the supernatant was collected and stored at 4 °C for later use.

#### 2.3.2 Synthesis of AgCDs

A mixture of 6 mL of prepared CDs, 3 mL AgNO_3_ (0.1 M), and 5 mL NaOH (0.1 M) was heated at 90 °C under magnetic stirring at 450 rpm for 50 min.^20^ After cooling to room temperature, the solution was subjected to centrifugation at 5000 rpm for 15 min, and the deposit was washed with DI water and ethanol, successively. Finally, the deposit was dissolved in deionized (DI) water and centrifuged at 5000 rpm for 15 min. The supernatant containing AgCDs was then recovered and stored at 4 °C for further use.

#### 2.3.3 Pretreatment of the Au electrode

Before using the Au electrode, the surface of the gold disc was polished with alumina (0.05 µm diameter), followed by ultrasonic cleaning with DI water and ethanol for two minutes at each step, successively. This electrode was subsequently cleaned electrochemically in a 1 M sulfuric acid (H_2_SO_4_) solution by polarizing the electrode for 60 s at −0.6 V and +1.5 V to form H_2_ and O_2_ bubbles on the electrode surface, respectively. Cyclic voltammograms (CV) were then recorded between −0.6 V and +1.5 V at 100 mV s^−1^ until reproducible cycles were obtained.^21^

#### 2.3.4 CDs/Au and AgCDs/Au electrode fabrication process

After cleaning, the electrode was immersed in a solution of CDs or AgCDs to design the CDs/Au and AgCDs/Au electrodes, respectively. This process was done for different times to optimize the designed electrode, which was finely dried at room temperature (28 °C) as suggested in the literature.^21^ The number of layers (number of successive immersion–drying cycles of the disc part of the electrode) was also optimized. The resulting CDs/Au or AgCDs/Au electrode was finally used in a Na_2_SO_4_ aqueous solution as the electrolyte for MG and LMG detection in the potential range from −0.1 V to +1.2 V at a scan rate of 50 mV s^−1^, using cyclic voltammetry (CV) and differential pulse voltammetry (DPV) techniques.

### 2.4 Electrochemical detection of MG and LMG

The response to MG and LMG on the bare and modified gold electrodes was studied using CV and DPV techniques. In a typical procedure of the detection of the mixture of MG and LMG, 50 mL solution of suitable Na_2_SO_4_ concentration was transferred to the electrochemical cell, and then an appropriate concentration of the total amount of MG and LMG solution was added. After homogenization, the CV was recorded within the potential range of −0.1 and +1.2 V (only the useful part is displayed) in the cathodic direction at a scan rate of 50 mV s^−1^. For calibration, different concentrations ranging from 3 pg mL^−1^ to 100 pg mL^−1^ of MG and LMG mixtures were prepared and used in the DPV technique. All DPV experiments were conducted between −0.1 V and +1.2 V at a scan rate of 50 mV s^−1^, a pulse time of 0.02 s, a sampling time of 0.05 s, a dwell time of 2 s, a staircase potential of 20 mV, and a pulse amplitude of 100 mV.

### 2.5 Application in real samples

The quantification of total amounts of MG and LMG in food samples was carried out using a DPV technique. One milliliter of MG/LMG from a 15 mL acetonitrile-based extract sample was added in 50 mL of 10^−4^ M Na_2_SO_4_ in electrochemical cell. The extraction process was carried out as described in the literature, with slight modifications.^22^ Briefly, 10 g of fish muscle (10 g sample) was minced and placed in a 50 mL centrifuge tube, into which 15 mL of acetonitrile and 1.5 g magnesium sulfate were added. The mixture was stirred at 250 rpm for 10 min in a laboratory oscillator (IS-RDD3, Crystal, USA) and then centrifuged at 5000 rpm for 5 min to collect the supernatant. Finally, 1 g of alumina was added to the supernatant and shaken again to remove lipids. The mixture was then centrifuged at 5000 rpm for another 5 min to collect the supernatant, and the MG and LMG contents were calculated using the obtained calibration equation. Unless otherwise stated, all experiments in this work were conducted in triplicate (*n* = 3), and the mean was used throughout this work.

## 3 Results and discussion

### 3.1 Characterization of the CDs, AgCDs and AgCDs/Au electrode

**Fig. 1:**
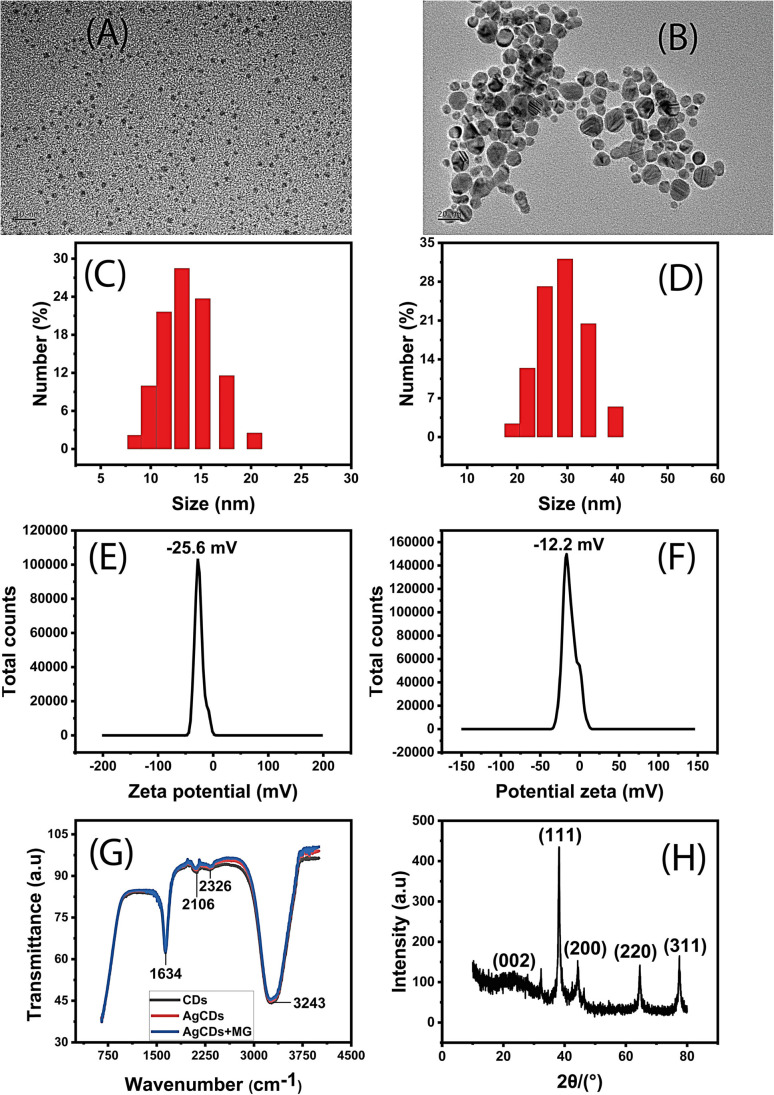
HRTEM images of (A) CDs and (B) AgCDs. DLS data of (C) CDs and (D) AgCDs. Zeta potential of (E) CDs and (F) AgCDs. (G) FT-IR spectra and (H) XRD spectrum of AgCDs.

The size and morphology of the synthesized CDs and AgCDs were analyzed by high-resolution transmission electron microscopy (HRTEM) and DLS (Fig. 1A–C). The results show that the CDs are spherical, well uniform, and dispersed with a particle diameter ranging from 4 nm to 10 nm (Fig. 1A). Similarly, AgCDs were also found to be well dispersed with a size between 5 and 16 nm (Fig. 1B). In addition, DLS measurements of CDs (Fig. 1C) show well-dispersed CDs with an average diameter of 14 nm, while AgCDs (Fig. 1D) exhibit good dispersion with an average diameter of around 29 nm. These results are consistent with the HRTEM results. It should be noted that the slight difference in values between these two techniques should be attributed to the fact that the DLS technique takes into account the solvation of the particles.

Moreover, zeta potential measurements have been carried out to scrutinize the surface charge (Fig. 1D and E). From these figures, both CDs and AgCDs indicate a negative charge on their surface. It can be observed that the CD surface charge (−25.6 mV) is two folds higher than that of AgCDs (−12.2 mV) with the same orders of conductivity of 0.554 mS cm^−1^ and 0.598 mS cm^−1^ for CDs and AgCDs, respectively. The decline of the surface charge from CDs to AgCDs may be due to the effect of Ag ions on the CDs during the synthesis of AgCDs in which the CDs can easily attract Ag ions due to their opposite charges. Meanwhile, the negative charge on the surface can cause repulsion between CDs, reducing the aggregation of these particles,^23^ and therefore, ensuring high stability. In order to know the different functional groups on the surface of CDs and to highlight the interactions that can occur between CDs and Ag^+^ ions during the AgCD synthesis, and between AgCDs and MG, the FT-IR spectra were recorded for CDs, AgCDs and a mixture containing MG and AgCDs (Fig. 1C). The FTIR spectra have a similar profile with a strong broadband absorption peak at 3243 cm^−1^, which implies the presence of O–H of alcohol and carbonyl, =C–H of alkene, N–H of amine and N–H and C=O of amide stretching, while the strong absorption peak at 1634 cm^−1^ confirms the presence of these alkenes, amines, alcohols, carbonyl groups and amides. However, the weak absorption peak at 2106 cm^−1^ and 2326 cm^−1^ may indicate the presence of alkyne functional groups. These results suggest the presence of amine, amide, alkene/alkyne, carbonyl, and alcohol functional groups on the surface of CDs and AgCDs. Furthermore, the lack of change in the spectra profile indicates that there is no obvious modification of the chemical functional groups on the surface of the CDs during the AgCD synthesis. Indeed, this effect can be explained by the fact that during the preparation of AgCDs, the Ag^+^ ions transformed two close amine functional groups into –N=N– and C–OH into –C=O on the surface of CDs, whose peaks appear almost at the same positions.^24^ However, the slight lower intensity of the different adsorption peaks of AgCDs and AgCDs-MG compared to those of CDs especially at 3243 cm^−1^ suggests that the reaction occurs between Ag and CDs (the effect between MG and AgCDs is almost negligible) through amide and carbonyl functional groups, and the used functional groups are negligible. Furthermore, the XRD results (Fig. 1D) show the facets of the face-centered cubic (fcc) crystal structure of silver (JCPDS, No. 04-0783) with the (111), (200), (220), and (311) planes. Moreover, the broad diffraction peak around 25° is the characteristic peak of carbon, confirming the formation of the AgCDs composite.^25^

**Fig. 2:**
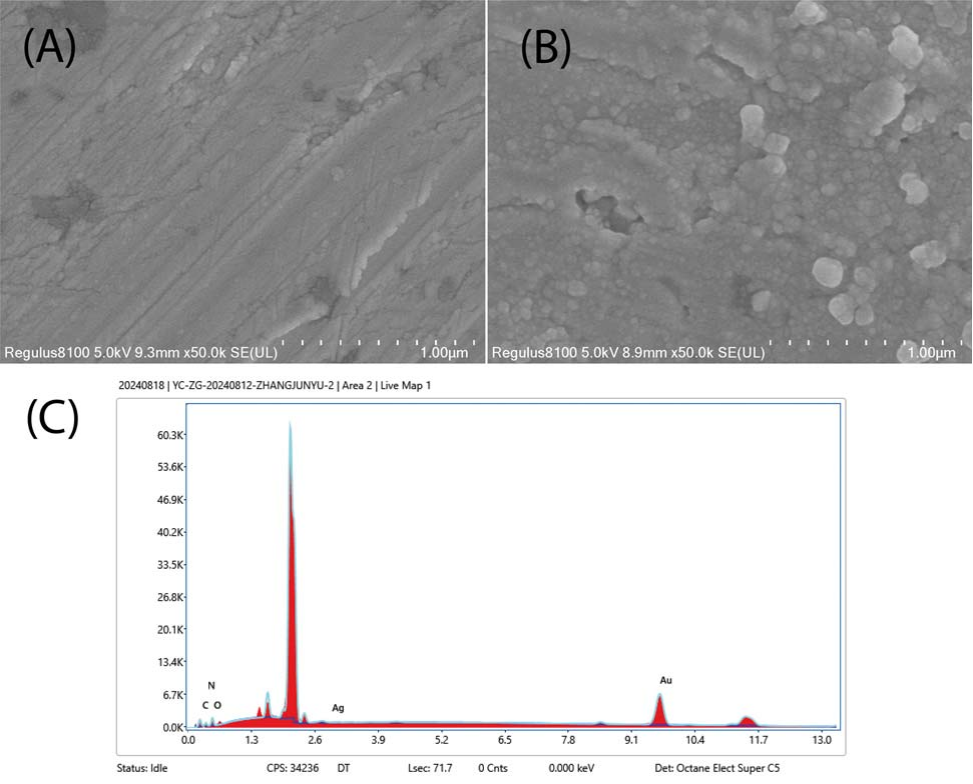
SEM images of the (A) bare gold electrode and (B) Au electrode after AgCDs deposition and (C) EDS spectrum of AgCDs/Au.

Besides, the active surface morphologies of the electrode were investigated to compare the bare and modified electrode surfaces. Fig. 2A and B show the SEM images of the surface morphologies of bare and modified gold electrodes with AgCDs, respectively. A noticeable change in the electrode surface morphology after the addition of AgCDs is observed with spherical AgCDs on the modified electrode surface. EDS analysis (Fig. 2C) confirms the presence of the observed chemical elements, with the FT-IR spectra, on the AgCDs/Au electrode, indicating the presence of AgCDs on the gold electrode active surface, as reported in the literature.^26^

### 3.2 Electrochemical characterization of the modified Au electrode

**Fig. 3:**
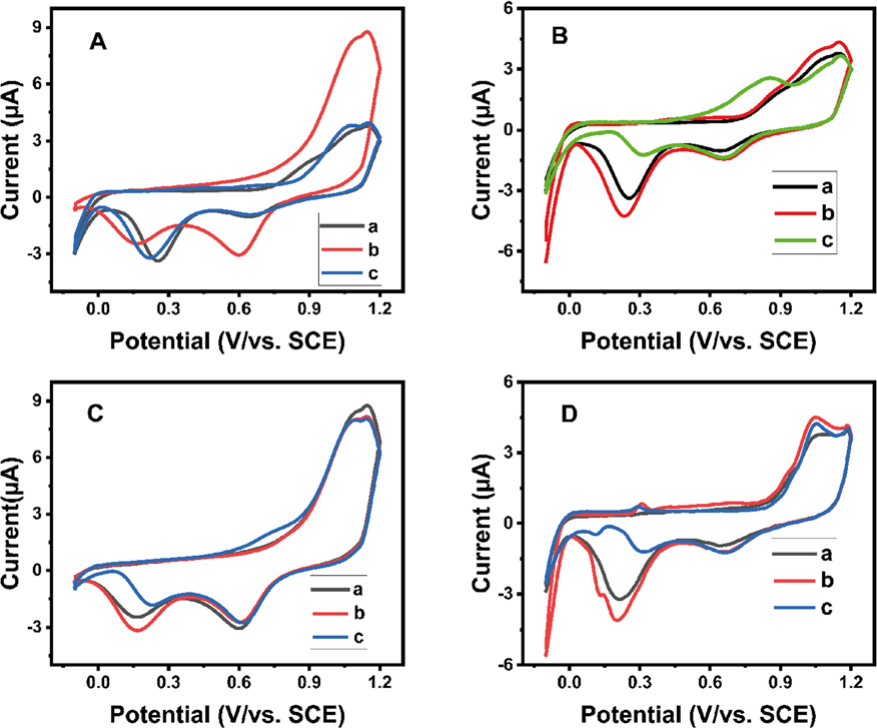
Cyclic voltammograms: (A) in the absence of MG and LMG on the (a) bare Au, (b) CDs/Au and (c) AgCDs/Au electrodes; (B) (a) without any contaminant, and in the presence of 5 ng mL^−1^ of (b) MG and (c) LMG on the bare Au electrode; (C) (a) without any contaminant, and in the presence of 5 ng mL^−1^ of (b) MG and (c) LMG on the CDs/Au electrode; and (D) (a) without any contaminant, and in the presence of 5 ng mL^−1^ of (b) MG and (c) LMG on the AgCDs/Au electrode in 0.03 M Na_2_SO_4_ at 50 mV s^−1^ scan rate.

The electrochemical behavior of bare gold, CD-modified gold and AgCD-modified gold electrodes in the absence and presence of MG and LMG was studied using the CV technique in 10^−4^ M Na_2_SO_4_ at a scan rate of 50 mV s^−1^ (Fig. 3A and B). The cyclic voltammetric responses (CV) of the supporting electrolyte showed a weak oxidation peak at 1.15 V and two reduction peaks at 0.654 V and 0.254 V at bare electrode (Fig. 3A(a)) and CDs-modified electrode (Fig. 3A(c)) are observed, while strong peaks at the peaks around 1.15 V and 0.654 V with CDsAgNPs-modified electrode (Fig. 3A(b)) are obtained along with weak reduction peak at 0.254 V. In general, the reduction peak at 0.254 V is the most affected (Fig. 3B–D). In all cases, its intensity increases in the presence of MG, but slightly less with LMG. Moreover, with the AgCD-modified Au electrode, in addition to the oxidation peak at 1.15 V and the two reduction peaks at 0.654 V and 0.254 V, another oxidation peak around 0.3 V and a reduction peak at 0.15 V (Fig. 3C(b and c)) were observed in the presence of MG and LMG. The appearance of these two new redox current peaks can be attributed to MG and LMG, validating the improvement of electron transfer on the fabricated electrode for the catalytic process of MG and LMG. This enhancement could be mainly due to the presence of a thin film of AgCDs on the surface of the Au electrode. These observations support the electrocatalytic nature of the AgCDs/Au electrode for the oxidation of MG and LMG. Indeed, AgCDs have several functional groups (amine and carbonyl functions) that can promote the oxidation and reduction process of MG and LMG onto the active catalytic hotspot, facilitating therefore the detection of MG and LMG on the fabricated electrode surface as suggested in the literature.^27^ Furthermore, since the characteristic oxidation peak at 0.3 V and the reduction peak around 0.15 V of both MG and LMG emerge at the same potential, MG and LMG can be detected as a single entity. To further investigate the underlying mechanism and the possibility of total amount detection, a mixture of 2.5 ng mL^−1^ of each compound is used for the rest of the cyclic voltammogram experiments.

### 3.3 Effect of the electrolyte (Na_2_SO_4_) concentration

The effect of the Na_2_SO_4_ concentration on the electrochemical response of the mixture of MG and LMG on AgCDs/Au was evaluated in the concentration range of 10^−5^ M–10^−1^ M (Fig. S1). The results show that the intensity of the MG and LMG mixture oxidation peak around 300 mV per SCE abruptly increases with the increase in the concentration of the electrolyte Na_2_SO_4_ (Fig. S1B) until 10^−4^ M and then decreases beyond. This decrease may be due to the activity of the ions in the electrolyte, which can behave as site hindrance for the MG and LMG oxidation reactions.^28^ Moreover, the intensity of the oxidation peak at 1150 mV/ECS decreases with the decrease in Na_2_SO_4_ concentration and disappears at 10^−4^ M, justifying that the optimized electrolyte concentration is 10^−4^ M. The concentration of the electrolyte is therefore set to 10^−4^ M for the remaining experiments.

### 3.4 Effect of the immersion time of the Au electrode in the AgCD solution

The effect of the immersion time of the Au electrode in the AgCD solution and the effect of droplet deposition of the colloidal AgCD solution onto the gold electrode surface were evaluated by CV for an immersion time varying between 5 min and 15 min (Fig. S2A) and for the number of drops ranging from 1 to 9 (Fig. S2C). No change in the profile of the graphs has been observed. However, an increase in the intensity of the MG and LMG mixture oxidation peak is observed for an increase in immersion time up to 10 minutes (Fig. S2B) and for the number of drops up to 8 (Fig. S2D) which then decreases beyond. This maximum at 10 minutes and for 8-drop deposition prove that increasing the immersion time or the number of drops improves the charge transfer, achieving a maximum of the conductivity-specific surface area of the modified AgCDs/Au electrode around 10 minutes or for an 8-drop deposition. The superposition of the voltammograms obtained after 10 minutes of immersion of the gold electrode and the deposition of 8 drops on the surface of the gold electrode (Fig. S2E) indicates that the peak intensity obtained by depositing 8 drops on the surface of the electrode is slightly greater than that obtained after 10 minutes of immersion of the gold electrode in the silver nanoparticle solution. However, the droplets are more unstable during drying, making the electrode less stable than the immersion electrode. For this purpose, the immersion technique was used.

### 3.5 Effect of the number of AgCD layer depositions

To find out the optimum number of deposits for a better detection for the total residues of MG and LMG mixture, the effect of the number of layers was examined by CV technique by fixing the immersion time at 10 min and the scanning speed at 50 mV s^−1^ in 10^−4^ M Na_2_SO_4_ in the presence of 2.5 ng mL^−1^ of each contaminant (MG and LMG) in the mixture (Fig. S3). As can be seen from this figure, the comparison of MG and LMG mixture oxidation peaks as a function of the number of deposits shows a maximum for the three-layer deposition, suggesting that the three-layer deposition is ideal for the fabrication of the AgCDs/Au electrode. This maximum indicates that the number of deposits increases the catalytic activity of AgCDs/Au, which can be explained by the fact that AgCDs decrease the electron transfer resistance till three layers.^29^ Therefore, the number of AgCD layers deposited on the Au electrode is set at three for the Au electrode immersion for the remaining experiments.

### 3.6 Effect of the scan rate and pH

**Fig. 4:**
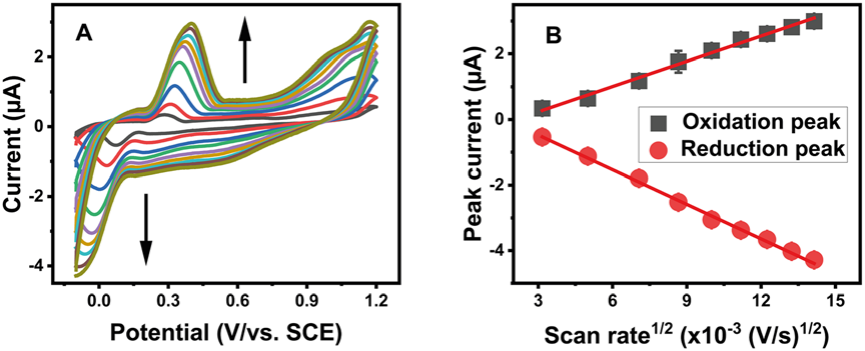
(A) Effect of the scan rate on the voltammetric response of the AgCDs/Au electrode in the presence of 2.5 ng mL^−1^ each of MG and LMG in the mixture. (B) Peak current intensity as a function of the square root of the scan rate.

The effect of the scan rate is one of the parameters that significantly affect the oxidation-reduction of various compounds. This effect on MG and LMG mixture oxidation and reduction peaks was evaluated at different scan rates ranging from 10 mV s^−1^ to 200 mV s^−1^ in a 10^−4^ M Na_2_SO_4_ solution in the presence of 2.5 ng mL^−1^ each of MG and LMG in the mixture (Fig. 4A). As shown, the peak currents increased linearly with *v*^½^ (inset of Fig. 4, or 4B), indicating the diffusion-controlled behavior.^21^ The linear regression equations can be expressed as follows:

1 *I*_pa_ = 0.255*v*^1/2^ (V s^−1^) − 0.5243 (*R*^2^ = 0.9923)

2 *I*_pc_ = −0.3488*v*^1/2^ (V s^−1^) + 0.5738 (*R*^2^ = 0.9966)

To avoid the likely overload during the experiments, the scan rate is fixed at 50 mV s^−1^ for all experiments.

**Fig. 5:**
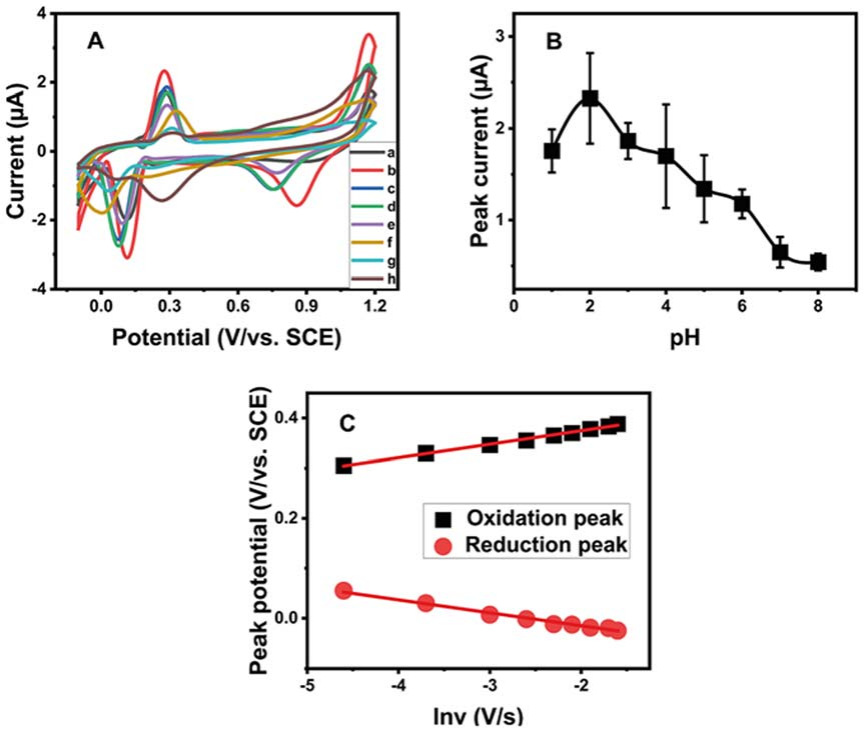
(A) Cyclic voltammograms of AgCDs/Au in the presence of 2.5 ng mL^−1^ each of MG and LMG in the mixture and in the Na_2_SO_4_ solution at different pH values of (a) 1, (b) 2, (c) 3, (d) 4, (e) 5, (f) 6, (g) 7, and (h) 8. (B) Oxidation current peak intensity as a function of pH of the Na_2_SO_4_ solution. (C) Oxidation and reduction potential peak intensity as a function of ln *v*.

**Scheme 1:**
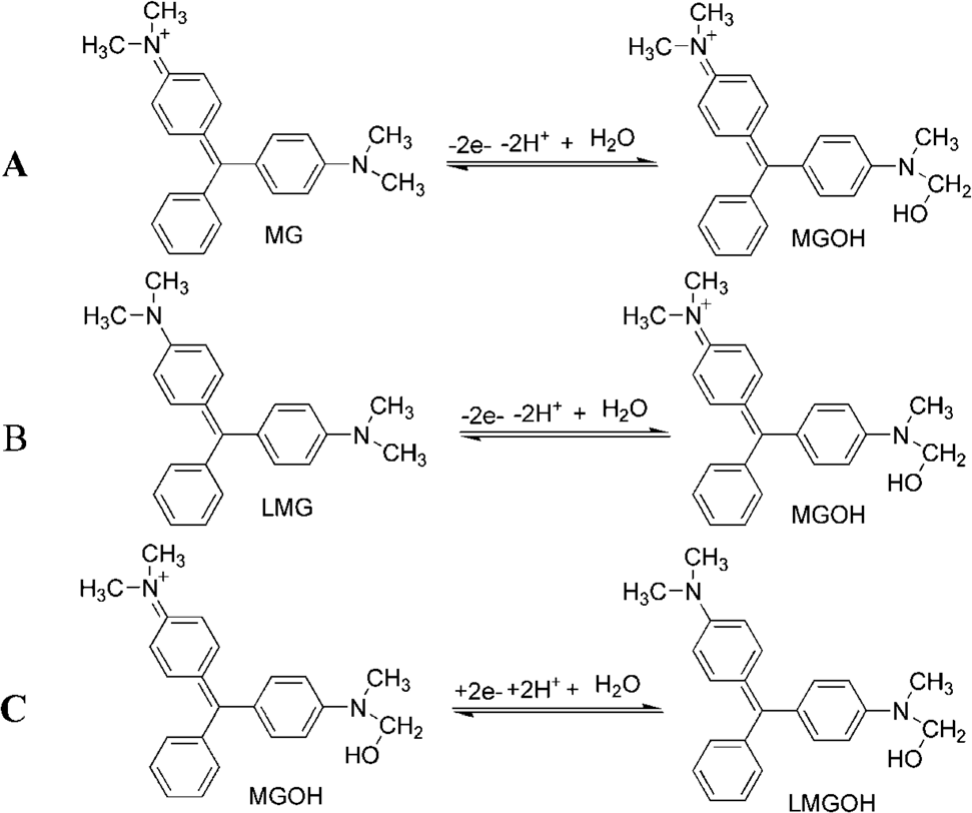
(A) and (B) Oxidation reactions of MG and LMG, respectively. (C) Reduction reaction of MGOH on the modified AgCDs/Au electrode.^32^

The pH effect of the electrolyte on the MG and LMG detection was also assessed in the pH range from 1 to 8 using the cyclic voltammetry technique. The pH of the medium was adjusted using NaOH and H_2_SO_4_ solutions. As shown in Fig. 5, an increase in the oxidation current peak was obtained in the pH range from 1 to 2, which then decreased with the pH from 2 to 8, with the highest current peak observed at pH 2. It has been reported that at pH 2, MG and LMG can oxidize electrochemically to give MGOH in the forward current wave, and in the reverse current, MGOH in turn reduces to LMGOH (Scheme 1).^30,31^ The decrease in the intensity of the oxidation peak observed above pH 2 could be explained by the fact that the higher the pH, the lower the MG peak due to the conversion of the MG form to the carbinol form, and hence, the lower the MGOH formation. Consequently, pH 2 was chosen for further experiments.

In parallel, based on the *E*_p_ = *f*(ln *v*) curve (Fig. 5C), the relationships between the potential peak and ln *v* are as follows:

3 *E*_pa_ = 0.027 ln *v* + 0.4283 (*R*^2^ = 0.9952)

4 *E*_pc_ = −0.0265 ln *v* – 0.0691 (*R*^2^ = 0.9903)

This follows eqn (5) and (6):^10^5
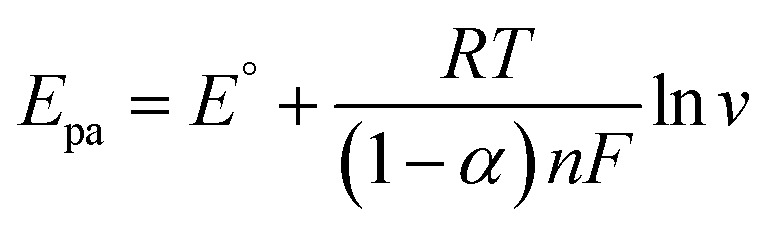
6
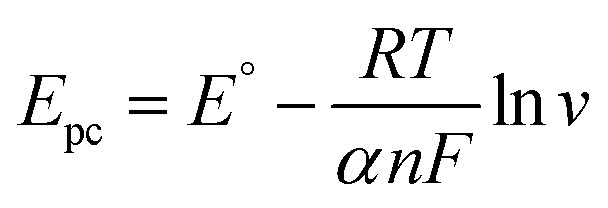
where *E*^°^ is the formal potential, *R* the universal perfect gas constant (8.314 J mol^−1^), *F* Faraday's constant (96 487 C mol^−1^), *T* the temperature in Kelvin (298 K), *v* the sweep rate in potential (V s^−1^) and *α* the charge transfer coefficient. Using Laviron's theory,^10^ the slope of *E vs.* ln *v* gives an electron transfer number of 2.

These results are in good agreement with previous works which stipulated that in highly acidic solutions, MG and LMG undergo two-electron oxidation and reduction, as explained in Scheme 1.^30,32^ Furthermore, the reduction peak observed during the oxidation for MG or LMG suggests that MG and LMG oxidation lead to the same product. This confirms the proposed mechanism equation. As the oxidation of MG and LMG formed the same reducing agent, this latter product can be used to detect the total amount of MG and LMG in a sample.

### 3.7 Determination of MG and AgCDs/Au sensitivity

**Fig. 6:**
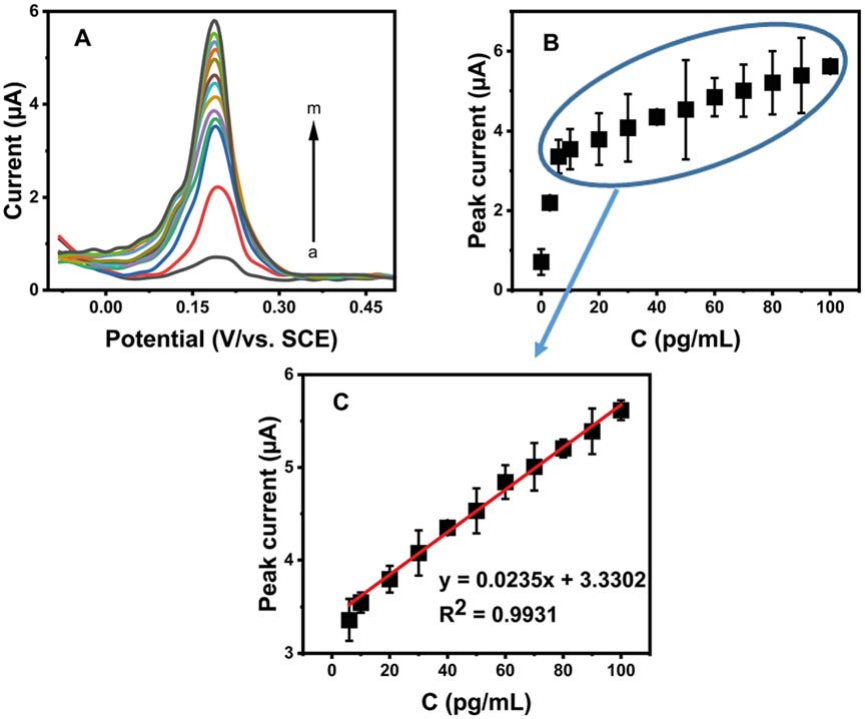
(A) DPV for different concentrations of the MG and LMG mixture in pg mL^−1^: (a) 0; (b) 3; (c) 6; (d) 10; (e) 20; (f) 30; (g) 40; (h) 50; (i) 60; (j) 70; (k) 80; (l) 90 and (m) 100 in 10^−4^ M Na_2_SO_4_ at pH 2. (B and C) Relationship between the maximum intensity of the oxidation peak current and the concentration of the MG and LMG mixture: calibration plot of the peak current against the concentration of the MG and LMG mixture.

The sensitivity of the AgCDs/Au electrode was assessed by studying the DPV response of MG and LMG mixture oxidation on the AgCDs/Au electrode (Fig. 6A) in 10^−4^ M Na_2_SO_4_ at pH 2. For simplicity, we calibrated the total mixture signal. As shown in Fig. 6B and C, good linearity ranging from 6 pg mL^−1^ to 100 pg mL^−1^ was obtained between the oxidation peak intensity and the MG and LMG mixture concentration with the associated linear equation:

7 *I*_(µA)_ = 0.0235*C* (pg mL^−1^) + 3.3302 (*R*^2^ = 0.9931)

The limit of detection and quantification calculated with the 3 s S^−1^ and 10 s S^−1^ relationships (where *s* is the standard deviation and S, the slope of the calibration curve (*n* = 10)) give around 4.95 pg mL^−1^ and 16.48 pg mL^−1^, respectively. This low detection limit indicates the high sensitivity of the fabricated electrode for the detection of the total amount of MG and LMG detection, and also suggests that this electrode can be used where monitoring of MG and LMG is required. As the values of limit of detection and quantification are lower than the recommended limits in foodstuffs, it could be important for ensuring food safety and environmental protection.

### 3.8 Average recovery using the fabricated electrochemical sensor

The reliability of the proposed method was assessed based on recovery studies using six different concentrations of MG and LMG in 10^−4^ M Na_2_SO_4_ at pH 2. The obtained recovery was approximately 99% (Table S1), demonstrating the excellent accuracy of this technique.

### 3.9 Selectivity of the AgCDs/Au electrode

In order to verify the selectivity of this approach, certain potential interferences in the organisms or environment were tested in 10^−4^ M Na_2_SO_4_ at pH 2. The experimental results are presented in Table S2. As can be seen, the presence of 10-fold concentrated Cu^2+^, Fe^2+^, Zn^2+^, NaCl, bovine serum albumin, cod liver oil, chloramphenicol (CAP), and 4-(dimethylamino)phenol hydrochloride (*p*-DAP) had low effects on the response of 10 pg mL^−1^ of the MG and LMG mixture (signal change is less than 5%). These results show recovery between 98% and 100%, demonstrating that this method has good selectivity.

### 3.10 Precision

The precision was evaluated at various concentration levels (8–55 pg mL^−1^). These tests were conducted in two different ways: intraday, which involved measuring the concentrations in the morning, noon, and evening throughout the same day, and interday, which allowed us to evaluate the method during a specific time of day over three days. Finally, we calculated the standard deviations. As can be seen, the relative standard deviations in both cases were less than 10% (Table S3). This high accuracy suggests that this electrochemical sensor can be used to monitor MG and LMG.

### 3.11 Reusability and stability of the manufactured electrochemical sensor

Electrode reusability and stability were tested daily over 10 days with 10 successive cycles in 10^−4^ M Na_2_SO_4_ at pH 2. As shown in Fig. S4, no obvious change in peak intensity or CV profile is observed for the MG and LMG oxidation on the AgCDs/Au electrode, indicating that the AgCDs/Au electrode is stable. This effect also indicates that the developed AgCDs/Au electrode can be reused several times and several days without any particular precautions, demonstrating that the coated electrode is robust and does not significantly degrade or foul after repeated oxidation/reduction cycles. In addition, for the reusability investigation, the same AgCDs/Au electrode was used for 10 days in solutions prepared daily with the same concentration of MG and LMG (three parallel experiments were conducted). After use, the electrode was washed successively with water and ethanol and left to dry at room temperature. As indicated in Table S4, no effective decrease in recovery was observed over the ten days for the same electrode (not more than 10% of the relative standard deviation was obtained). These poor effects on MG and LMG mixture determination indicate that the developed AgCDs/Au electrode can be reused several times and several days without any particular precautions.

### 3.12 Application of the fabricated electrochemical sensor in real samples

The developed electrochemical sensor was evaluated on sheatfish, carp, red carp and catfish in 10^−4^ M Na_2_SO_4_ at pH 2. Table 1 shows the levels of total MG and LMG in these food matrices. The total MG and LMG concentrations obtained vary from 669.51 to 1484.06 pg g^−1^ for sheatfish, 497.51 to 1179.72 pg g^−1^ for carp, 324.3 to 1093.59 pg g^−1^ for red carp and 625.58 to 1059.23 pg g^−1^ for catfish. These results showed that the total amount of MG and LMG remained non-negligible (above 2 µg kg^−1^ regulatory limit), indicating the illegal use of MG. These results are in good agreement with most studies reported in the literature.^39^ These findings suggest that despite regulations, malachite green is still being used. Therefore, increasing awareness and strengthening efforts may be necessary to fully eliminate its use in aquaculture.

**Table 1:** Assessment of the MG and LMG contents in food products

Food products	Municipality			
	Adjame zone		Cocody zone	
	Min–max^a^ (pg g^−1^)	Standard deviation	Min–max^a^ (pg g^−1^)	Standard deviation
Sheatfish	673.88–877.09	0.37	669.51–1484.06	0.82
Carp	497.51–923.06	0.71	767.74–1179.72	0.64
Red carp	625.18–11093.59	0.42	324.3–527.81	0.11
Catfish	836.25–1059.23	0.34	625.58–710.77	0.05

a Minimum–maximum.

### 3.13 Comparison with other electrochemical methods used for MG and LMG detection

The comparison (Table 2) shows that our sensor's LOD is among the lowest reported for MG/LMG detection methods, and well below the regulatory threshold. In contrast, conventional methods like LC-MS/MS have an LOD in the ng mL^−1^ range, and even the limit of detection of ELISA is around 50 pg mL^−1^. Due to its simplicity, selectivity, and LOD well below the recommended limits, the proposed portable sensor could be one of the best options for the on-site monitoring of MG and LMG.

**Table 2:** Comparison with other electrochemical methods for the determination of MG and LMG

Method	Linear range (pg mL^−1^)	Detection limit (pg mL^−1^)	Ref.^a^
HPLC	(2–1000 µg L^−1^) 2 × 10^3^ − 10^6^	(1.7 µg L^−1^) 1700	33
Immunochromatographic (MG & LMG)	Not reported	(MG: 0.221 µg L^−1^) 221 (LMG: 0.214 µg L^−1^) 214	34
A colorimetric aptasensor based on RNA and gold nanoparticles	(20–300 nM) 7.3 × 10^3^ − 109.5 × 10^3^	(15.95 nM) 5.8 × 10^3^	12
Fluorescence-based CdTe-MIP/SiO_2_	(0.01–20 µmol L^−1^)	(3.7 nmol L^−1^)	4
A fluorescent aptasensor based on the photochromic aptamer switch assay	Not reported	(2 µM) 728.2 × 10^3^	35
A ratiometric fluorescent sensor with molecularly imprinted mesoporous microspheres	(27.4 nM – 137 µM) 10 × 10^3^ − 49992.8 × 10^3^	(17 nM) 6.2 × 10^3^	36
CeO_2_/Nafion/GCE electrode	(1.0–10 µM) 364.9 × 10^3^ − 3649.1 × 10^3^	(1025 µM) 374.03 × 10^3^	2
SERS	(10 fM – 100 µM) 0.00365 − 36.5 × 10^6^	(2 fM) 0.729	37
MWCNTs-PEI/GCE electrode	(0.01–6.0 µM) 3649.11 × 10^3^ − 2189466 × 10^3^	(2.58 nM) 941.47	38
HDPB/ABPE electrode	(0.02–40 µM) 7.3 × 10^3^ − 14596.44 × 10^3^	(4.0 × 10^−3^ µM) 1.46 × 10^3^	5
AgCDs/Au electrode	6–100	4.95	This work

a Ref.: References.

Therefore, this work is important for countries with no access to traditional lab analysis of food safety and quality, where MG and LMG monitoring is required.

## 4 Conclusions

In this study, a fabricated AgCDs/Au electrode with a portable workstation system was used for the detection of the total amount of MG and LMG. The results show that the AgCDs/Au electrode exhibits good activity for the oxidation of MG and LMG mixture in a Na_2_SO_4_ medium. This electrocatalytic activity suggests a synergistic effect of CDs, AgNPs and Au on the oxidation of MG and LMG mixture on the AgCDs/Au electrode. In addition, this AgCDs/Au electrode has proven to be stable, and could be reused several times with satisfactory results. The detection limit can be as low as 4.95 pg mL^−1^, which is comparable to the LOD of traditional techniques, which is around 2 µg kg^−1^. This detection limit is below the recommended limits in foodstuffs, indicating that this technique can be used where the monitoring of MG and LMG is of critical importance. In addition, the method is reliable, with good recoveries and accuracy. All these results indicate that the AgCDs/Au method is ideal for the electrochemical detection of MG and LMG, with several advantages such as on-site measurement, non-use of electrical energy, and simple procedures and setup, suggesting that the AgCDs/Au sensor is a promising tool for the ultrasensitive on-site monitoring of MG and LMG residues in aquaculture products. Furthermore, the miniaturization of the whole electrochemical tools and the fact that no electric current is required make this process suitable for on-site measurements.

Future work should focus on further validating this sensor in diverse real-world conditions and exploring its application to other contaminants.

## Author contributions

All the authors contributed equally.

## Conflicts of interest

There are no conflicts to declare.

## Supplementary Material

RA-016-D6RA01261B-s001

## Data Availability

No primary research results, software or code have been included and no new data were generated or analysed as part of this review. Supplementary information (SI) is available. See DOI: https://doi.org/10.1039/d6ra01261b.
