# Supplementary material for: Carbon dot-modified silver nanoparticle electrochemical sensors for the ultrasensitive detection of total malachite green and leucomalachite green residues in fish
Source: RSC Adv. 2026 Mar 30;16(19):17149–57. doi: 10.1039/d6ra01261b (PMC13034132; doi:10.1039/d6ra01261b)
Supplement: RA-016-D6RA01261B-s001 [file RA-016-D6RA01261B-s001.pdf]

SUPPORTING INFORMATION

# Carbon dot-modified silver nanoparticle electrochemical sensor for ultrasensitive detection of total malachite green and leucomalachite green residues in fish.

Pomi Bi Boussou Narcisse[a], Aka Alla Martin[a], Essy Kouadio Fodjo*[a], Guangxin Yang[b], Cong Kong[b], Zhen Gu[c], Koffi Koffi Kra Sylvestre[a], Irié Bi Irié Williams[a]

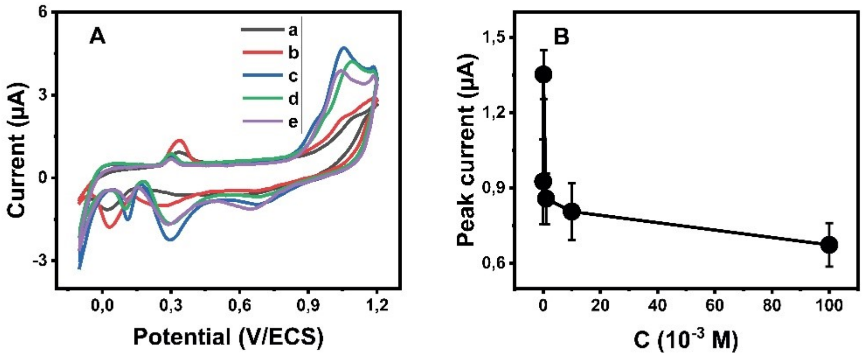


**Fig. S1** A) Cyclic voltammograms of AgCQDs/Au in the presence of (a) $10^{-5}$ M; (b) $10^{-4}$ M; (c) $10^{-3}$ M; (d) $10^{-2}$ M and (e) $10^{-1}$ M of $Na_2SO_4$ with 2.5 ng/mL of each MG and LMG in their mixture at 50 mV/s. B) Intensity of the oxidation current peak as a function of $Na_2SO_4$ concentration.

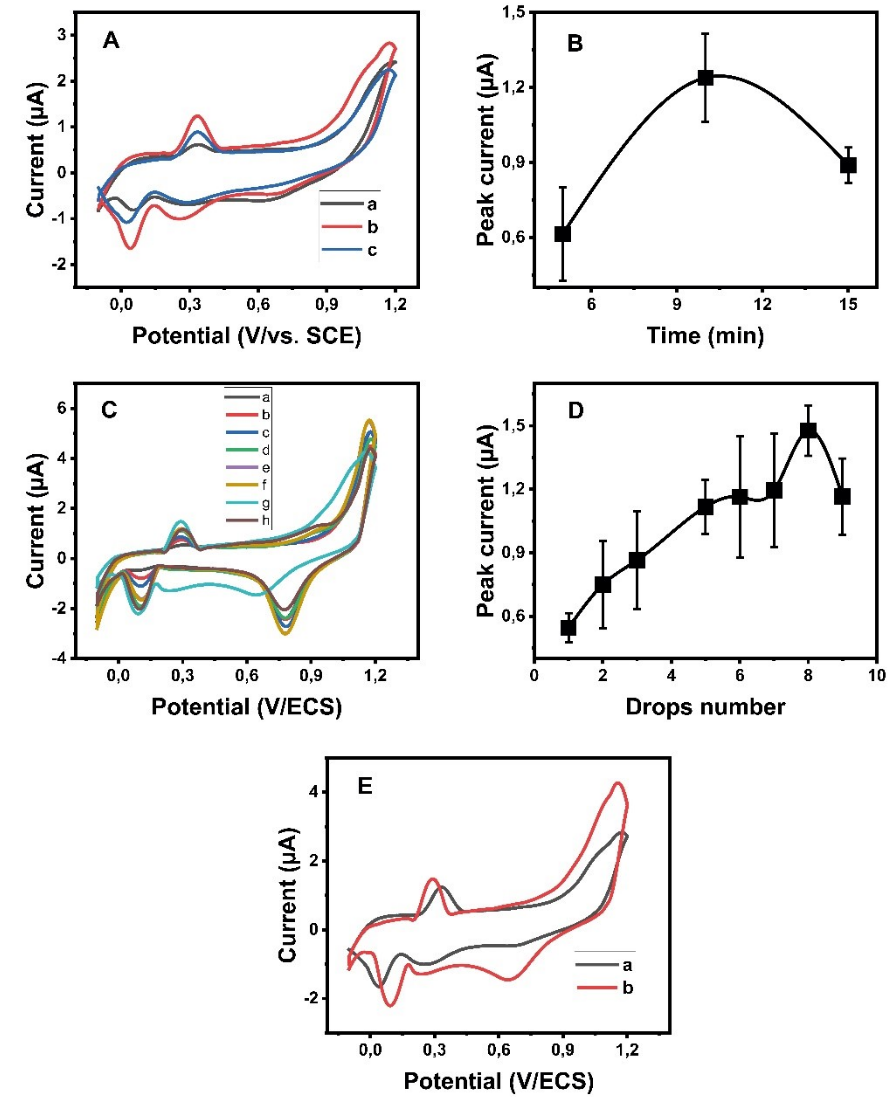


**Fig. S2:** A) Cyclic voltammograms recorded in $10^{-4}$ M $Na_2SO_4$ solution at 50 mV/s in the presence of 2.5 ng/mL of each MG and LMG in their mixture at different immersion times of the Au electrode in the colloidal solution of AgCDs. B) Peak current intensity as a function of immersion time (a) 5, (b) 10 and (c) 15 min. C) Cyclic voltammograms recorded in $10^{-4}$ M $Na_2SO_4$ solution at 50 mV/s in the presence of 2.5 ng/mL of each MG and LMG in the mixture for different number of drops of colloidal AgCDs solution on the surface of the Au electrode. D) Peak current intensity as a function of the number of droplets deposited from (a) 1 drop to (h) 8 drops. E) Superposition of the voltammograms obtained for (a) 10 min of immersion and (b) deposition of 8 drops.

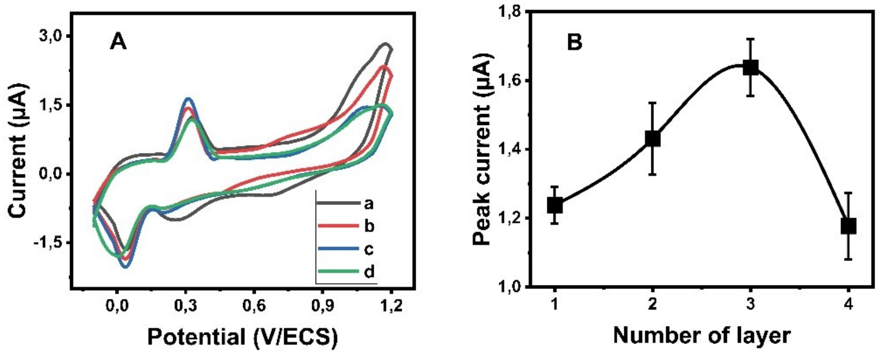




This journal is © The Royal Society of Chemistry 20xx

Please do not adjust margins



**Fig. S3** A) Cyclic voltammograms of the AgCDs/Au electrode obtained from deposition of a) one layer, b) two layers c) three layers and d) four layers of AgCDs on the Au electrode in a $10^{-4}$ M $Na_2SO_4$ solution at 50 mV/s containing 2.5 ng/mL of each MG and LMG in the mixture. B) Peak current intensity of the MG and LMG mixture oxidation as a function of the number of layers.

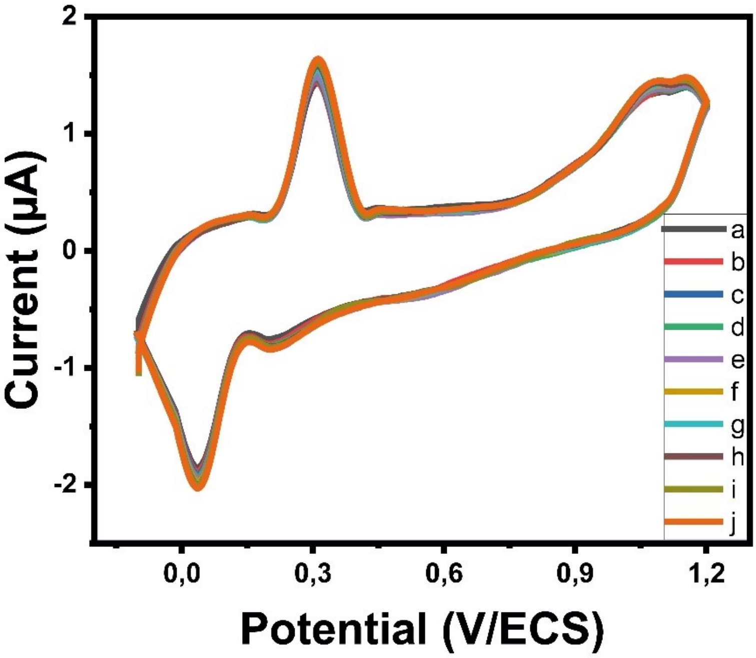


**Fig. S4** Stability study of the AgCDs/Au electrode.

**Table S1** Recovery using the fabricated sensor (n=3)

| Added (ng/mL) | Found (pg/mL)[a] | recovery (%) |
|---|---|---|
| 6 | 5.88 | 98±1.5 |
| 10 | 9.92 | 99.2±2 |
| 15 | 14.93 | 99.5±3.6 |
| 25 | 24.96 | 99.4±5.7 |
| 35 | 34.96 | 99.8±2.1 |
| 45 | 44.92 | 99.8±6.0 |
| 55 | 54.95 | 99.9±8.1 |

[a] Average value of recovered MG concentration.

**Table S2** Recovery rate of 10 pg/mL MG in the presence of 100 pg/mL of the selected interfering compounds (n=3).

| Compounds | C(pg/mL)[a] | C(pg/mL)b | Recovery (%) |
|---|---|---|---|
| MG | 10 | 10 | 100 |
| CAP | 10 | 9.98 | 99.8 |



Please do not adjust margins

Please do not adjust margins



| | | | |
|---|---|---|---|
| p-DAP | 10 | 9.98 | 99.8 |
| Bovine serum albumin | 10 | 9.95 | 99.5 |
| Cod liver oil | 10 | 9.92 | 99.2 |
| $CuSO_4$ | 10 | 9.94 | 99.4 |
| $FeSO_4$ | 10 | 9.97 | 99.7 |
| $Zn(NO_3)_2$ | 10 | 9.95 | 99.5 |
| NaCl | 10 | 9.87 | 98.7 |

$C^a$: Concentration added. $C^b$: Concentration found

**Table S**3: Intra-day and Inter-day studies (n=3).

| Concentration added (pg/mL) | Concentration found (pg/mL) | | RSD (%) (n=3) | |
|---|---|---|---|---|
| | Intra-day | Inter-day | Intra-day | Inter-day |
| 8 | 7.93 | 7.88 | 4.9 | 9.29 |
| 15 | 14.86 | 14.91 | 8.5 | 4.36 |
| 25 | 24.82 | 24.91 | 6.66 | 9.61 |
| 35 | 34.92 | 34.89 | 3.06 | 0.57 |
| 45 | 44.83 | 44.82 | 9.17 | 3.51 |
| 55 | 54.9 | 54.89 | 7.00 | 2.52 |

**RSD**: Relative Standard Deviation.

**Table S4:** Stability of the AgCDs/Au electrode, 15 pg/mL MG and LMG each day.

| Day | added (pg/mL)[a] | Found (pg/mL)[b] | Recovery (%) | RSD (%) (n=3) |
|---|---|---|---|---|
| 1 | 15 | 14.92 | 99.47 | 3.05 |
| 2 | 15 | 14.87 | 99.13 | 2.52 |
| 3 | 15 | 14.89 | 99.27 | 1.00 |
| 4 | 15 | 14.88 | 99.20 | 2.65 |
| 5 | 15 | 14.86 | 99.06 | 2.52 |
| 6 | 15 | 14.84 | 98.93 | 2.08 |
| 7 | 15 | 14.85 | 99.00 | 1.53 |
| 8 | 15 | 14.82 | 98.8 | 1.50 |
| 9 | 15 | 14.79 | 98.60 | 3.10 |
| 10 | 15 | 14.81 | 98.73 | 4.58 |

 This journal is © The Royal Society of Chemistry 20xx

Please do not adjust margins
